# Canine circovirus: An emerging or an endemic undiagnosed enteritis virus?

**DOI:** 10.3389/fvets.2023.1150636

**Published:** 2023-04-17

**Authors:** Diana Gomez-Betancur, Diana S. Vargas-Bermudez, Sebastian Giraldo-Ramírez, Jairo Jaime, Julian Ruiz-Saenz

**Affiliations:** ^1^Grupo de Investigación en Ciencias Animales—GRICA, Facultad de Medicina Veterinaria y Zootecnia, Universidad Cooperativa de Colombia, Bucaramanga, Colombia; ^2^Universidad Nacional de Colombia, Facultad de Medicina Veterinaria y de Zootecnia, Centro de investigación en Infectología e Inmunología Veterinaria (CI3V), Sede Bogotá, Bogotá, Colombia; ^3^Facultad de Medicina Veterinaria y Zootecnia, Fundación Universitaria Autónoma de las Américas, Medellín, Colombia

**Keywords:** CanineCV, canines, enteritis, diarrhea, coinfection

## Abstract

Canine Circovirus (CanineCV) belongs to the family *Circoviridae*. It is an emerging virus described for the first time in 2011; since then, it has been detected in different countries and can be defined as worldwide distribution virus. CanineCV infects domestic and wild canids and is mainly related to hemorrhagic enteritis in canines. However, it has been identified in fecal samples from apparently healthy animals, where in most cases it is found in coinfection with other viral agents such as the canine parvovirus type-2 (CPV). The estimated prevalence/frequency of CanineCV has been variable in the populations and countries where it has been evaluated, reaching from 1 to 30%, and there are still many concepts to define the epidemiological characteristics of the virus. The molecular characterization and phylo-evolutive analyses that allow to postulate the wild origin and intercontinental distribution of the virus. This review focuses on the importance on continuing research and establish surveillance systems for this emerging virus.

## 1. Introduction

The *Circoviridae* family comprises nonenveloped viruses with a diameter of 15–25 nm and a single-stranded circular DNA genome of less than 2,500 nt in length. According to the international committee on virus taxonomy (ICTV^1^), two genera have been recognized within this family: *Circovirus* and *Cyclovirus* (1–3). Within the genus *Circovirus*, almost 50 species have been identified that infect birds and mammals (1), including the clinically relevant beak and feather disease (BFDV), goose circovirus, pigeon circovirus, canary circovirus, porcine circovirus and the most recently recognized canine circovirus (CanineCV) (4–6).

CanineCV has a covalently closed single-stranded DNA genome of 2062–2063 nt (7, 8). It has an ambisense genome with two main ORFs arranged inversely; ORF1 encodes the viral replicase gene (Rep) (nt 1–912) necessary for the initiation of the replication cycle, and ORF2 encodes the capsid protein (Cap) (nt 1,116–1928), which participates in the immune response of the host (1, 7). The Cap and Rep genes of CanineCV share, respectively, between 25 and 50% identity with the other known animal circoviruses (2). A third ORF (ORF3) has been proposed in samples from Thailand; this ORF is located between nt 470 and 889 in the antisense region in ORF1 (5). Studies in porcine circovirus type 2 (PCV2) relate ORF3 to a protein associated with viral pathogenesis and the induction of apoptosis; however, in the case of CanineCV, its function is not yet being clarified (5, 9).

## 2. Virus replication

**Figure 1:**
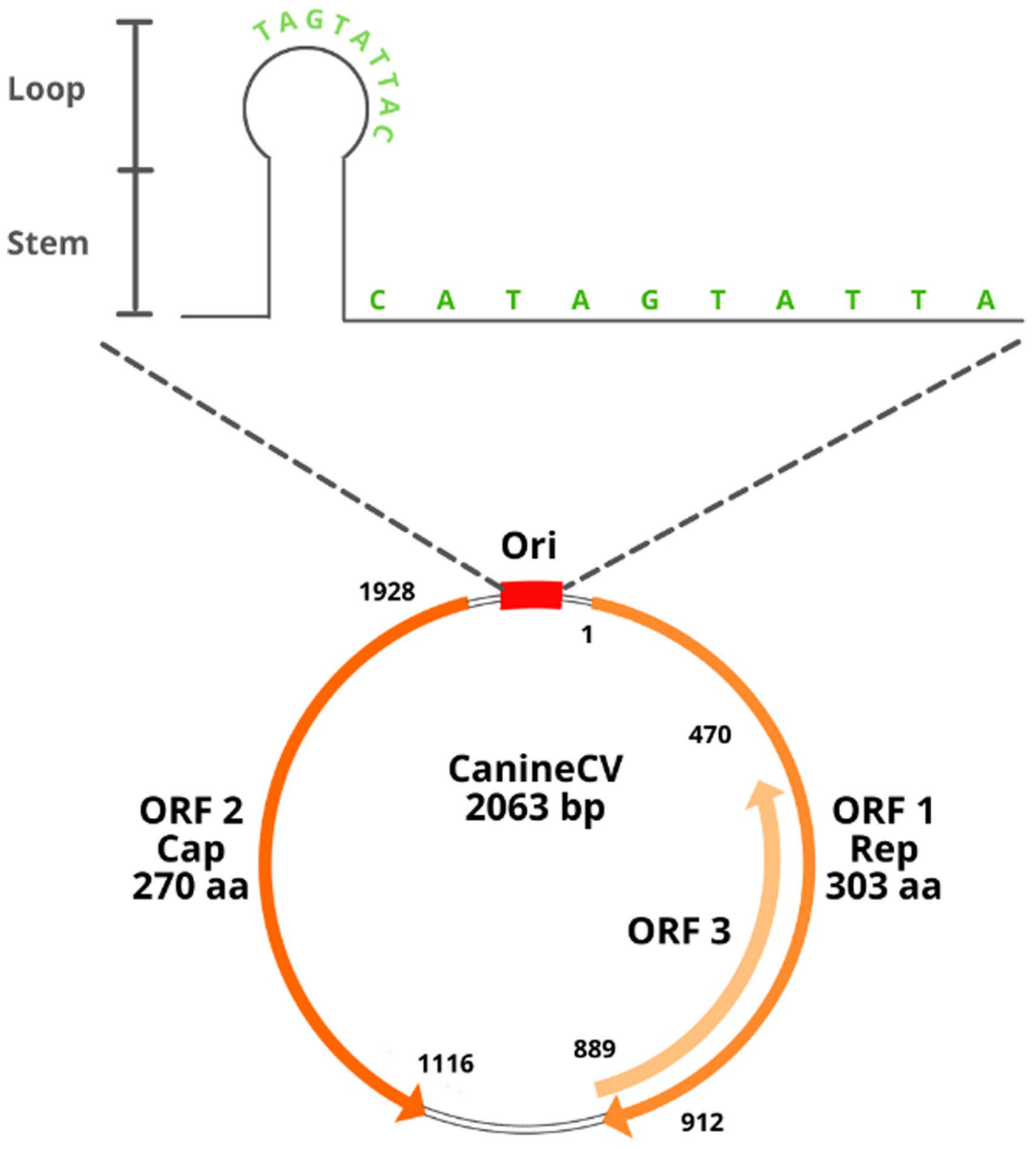
Structural diagram of the CanineCV genome. See text for references.

As with other circoviruses, CanineCV has 2 noncoding intergenic regions. The first corresponds to the 5′-intergenic region of 135 nt located between the start codons of the 2 ORFs and comprises the origin of replication (Ori). The latter is a thermodynamically stable conserved stem–loop structure with a characteristic sequence of 9 nt for the initiation of replication (TAGTATTAC), a palindromic sequence of 12 nt pairs and an open loop of 10 nt (CATAGTATTA) (2). The second intergenic region is the 3′ region of 203 nt, located between the termination codons of the two main ORFs (Figure 1). These highly conserved sequences in circoviruses are associated with rolling circle replication (2, 10).

The rolling circle mechanism constitutes the process of replication of circular DNA genomes. It has also been described in plasmids, plant viruses and other families of animal viruses, such as *Geminiviridae* and *Nanoviridae*. For circoviruses, the study model has been mainly that of porcine circovirus (PCV). Due to the reduced size of the viral genome that limits its coding capacity, this type of replication depends largely on host cell factors and requires cellular enzymes expressed during the S phase of the cell cycle (11, 12). It is estimated that the virus uses glycosaminoglycans as binding receptors to target cells and penetrates the cell by clathrin-mediated endocytosis (9). The ssDNA genome is transported to the nucleus, where it is converted to a supercoiled intermediate double strand that will serve as a template to generate viral DNA. In *Geminivirus* and *Nanovirus* species, the primer for the negative strand is composed of DNA or RNA with multiple 5′ ribonucleotides or RNA synthesized by the host after infection; however, the primer for the negative strand in circoviruses has not yet been determined (12).

**Figure 2:**
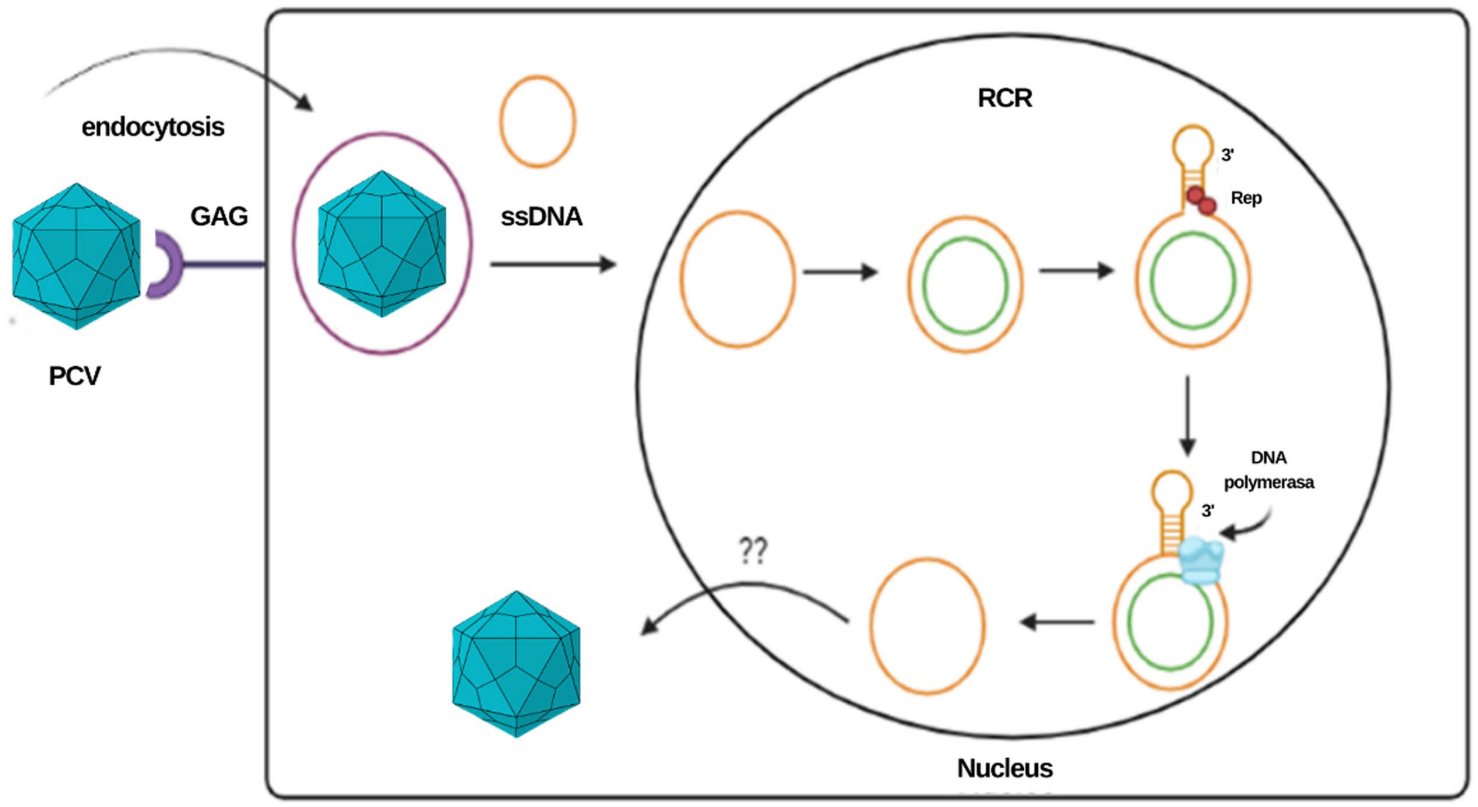
Representation of the PCV replication cycle and the rolling circle mechanism. This same mechanism is proposed for the CanineCV.

Once the double strand of the genome is formed, an initiation complex composed of Rep and Rep’ proteins that have endonuclease activity is attached to the stem–loop structure to cleave the loop, generating a free 3’OH end to which cellular DNA polymerase binds to initiate replication. When a unit of the genome has replicated, the Rep complex closes the loop covalently, leading to the release of a positive circular single-stranded parent molecule and a circular double-stranded DNA molecule, the latter composed of a hybrid of a negative parental strand and a positive strand newly synthesized (11, 12). The single-stranded DNA molecule can be encapsulated or involved in a second replication cycle. In *in vitro* studies in PCV, two different pathways have been suggested for the release of the virus from the cell at the end of the replication cycle: the first is the budding of groups of virions, and the second is cell lysis by apoptosis; however, the *in vivo* mechanism is still poorly understood [Figure 2; (11, 13)].

The “melting-pot” model has been proposed to explain the formation of the double-stranded intermediary. In this model, Rep proteins bind to palindromic sequences, generating a sphere of instability known as a melting pot. In this environment, the four inverted repeat strands present a “fused” state since there is an absence of hydrogen bonds between the positive and negative genomes. In this structure, not any double helix formation can be maintained, but the four strands remain very close to each other, juxtaposed in a tertiary structure that allows a possible exchange between the template strands during the synthesis of the genome, which provides two templates for the synthesis of the palindromic sequences during the initiation and termination of replication (11, 13). The availability of two template chains during the synthesis of the main chain contributes to the flexibility and the increase in the frequency of mutations in the Ori; however, the correction mechanism inherent to the melting pot allows mutations in the palindromic sequences to be eliminated, corrected, or regenerated through the mechanism of template change, allowing the integration of the nt sequence in the Ori (11).

## 3. Molecular epidemiology

CanineCV was identified for the first time in 2011 by Kapoor et al. (2) in serum samples from six of 205 clinically healthy dogs, confirming the first non-porcine circovirus to infect mammals (2, 4, 6). From this first report and to date, different studies of detection, genetic sequencing and phylogenetic analysis have been carried out in different countries worldwide, both in domestic dogs and in wild canids. The overall objective of such investigations has been mainly to establish the circulation of CanineCV at the demographic level in the region or area (2, 4). Virus detection studies have been based on molecular techniques including conventional and quantitative PCR (qPCR) (14, 15). Virus isolation has been unsuccessful. In MDCK cells infected with the CanineCV D1056 strain from Brazil only DNA was detected by qPCR in low amounts during the inoculation phase perhaps due to the amount of viral DNA found by qPCR for CanineCV was low (16). The same negative result has been reported by using Italian samples in MDCK and D17 cells (8). However, it is well known that most of the circoviruses such as PCV, are generally considered non-cytopathogenic and the low growth efficiency of PCVs in cultured cells has become a major obstacle to the establishment of diagnostic methods (17). Nevertheless, few reports have showed the cytopathic effect of PCV2 in specific cell lines (18, 19).

CanineCV is defined as a worldwide distribution agent since its circulation has been reported in 4 of 5 continents. The phylogenetic analyses of the strains reported to date have been prepared based on the complete genome and with the nucleotide sequences or concatenated amino acids of the Rep and Cap proteins (3, 4, 7). Multiple effort has been made to stablish a classification system that helps to understand virus origin and evolution. Sun et al. in China in 2018 based the phylogenetic analysis of the detected CanineCV strains on ORF2 sequences and proposed a classification into two groups: CanineCV-1 and CanineCV-2 (10). Subsequently, it was reported that the existing strains of CanineCV at the global level could be divided into four different groups: CanineCV-1, which includes strains from Italy, four from the United States, two from Germany, one from Argentina, and one from China. CanineCV-2 group strains from China, of which three were detected in 2014, 13 in 2015 and 24 in 2017. CanineCV-3 includes strains from China identified between 2014 and 2015, and CanineCV-4 contains a strain from the United States. three strains from the province of Guangxi and included the five strains detected in their research. The authors indicate that to differentiate the 4 groups, it is possible to use polymorphisms of individual nucleotide sequences (20). On the other hand, in the analysis conducted by Piewbang et al. in 2018 (5), it was concluded that the CanineCV sequences detected in their study were different from the majority of the sequences reported and were mainly related to the UCD3-478 strain from the United States. Additionally, the authors highlight that the phylogenetic trees based on the Rep and Cap genes of the CanineCV genomes circulating in Thailand are divergent and reveal uneven patterns when comparing both genes, which is associated with recombination events (5).

**Figure 3:**
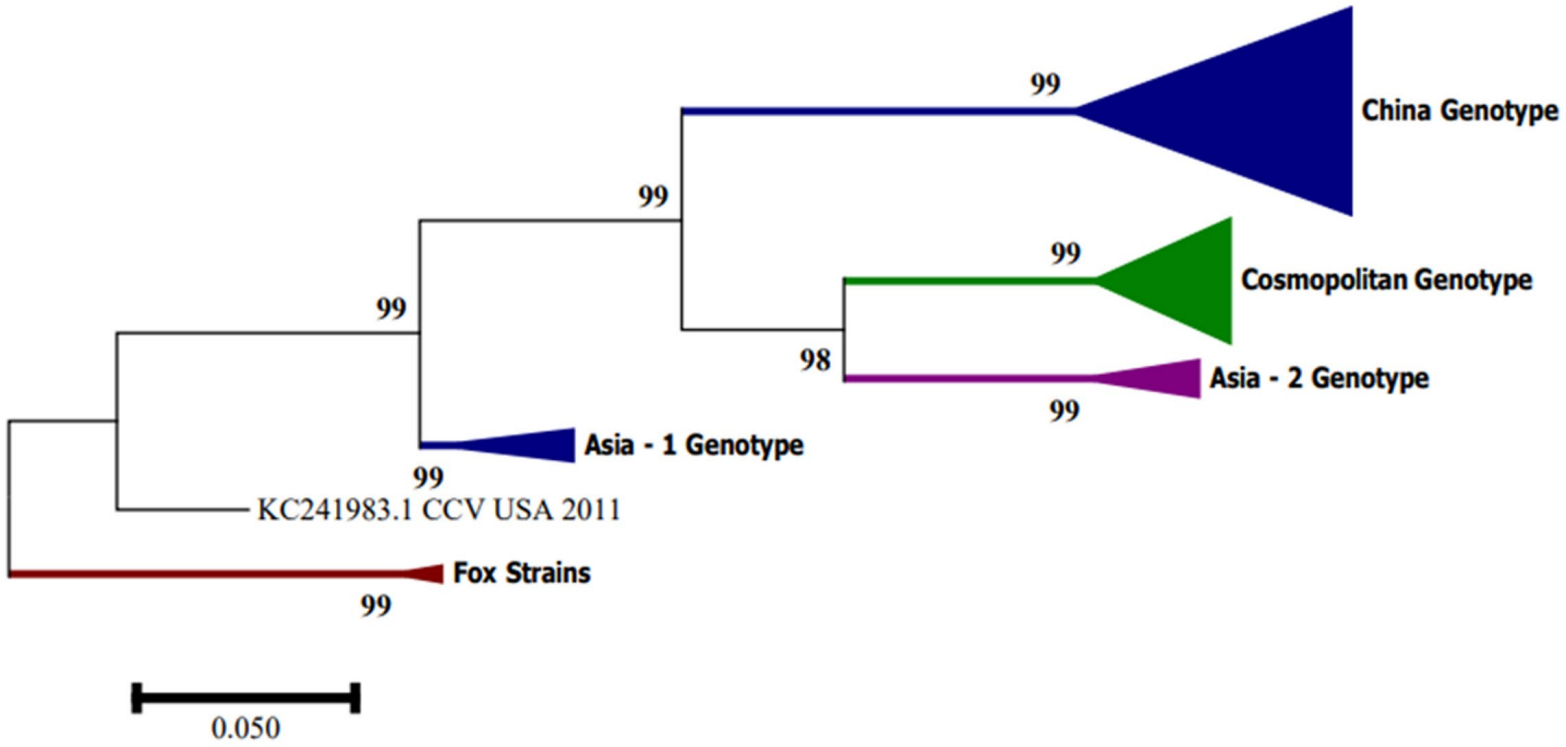
Phylogenetic tree based on complete CanineCV genome sequences. The phylogenetic analysis was performed using MEGA 7.0 for Windows by maximum likelihood with 1,000 bootstrap replicates and the 3-parameter Tamura nucleotide substitution model.

In South America, CanineCV has been detected in Argentina, Brazil and Colombia. Kotsias F et al. (21) detected the UBA-Baires strain of CanineCV in an outbreak of enteric disease in puppies between 2014 and 2015 in Argentina. Phylogenetic evaluation of whole genome sequences showed that this strain is grouped with viruses from Europe, mainly from Italy, and differed considerably from strains reported in the United States and China (21). In contrast, strain D1056 reported in Brazil by Cruz et al. (16) showed high similarity (<80%) with the NY214 strain reported in the United States. Giraldo-Ramírez et al. Colombia confirmed the separation of CanineCV strains into four different genotypes, designating clades according to geographical distribution and with the new sequences reported as follows: Genotype China includes sequences found in China, Genotype Asia-1 and Asia-2 include sequences from Thailand and China, and Cosmopolitan Genotype contains sequences from Italy, Germany, China, the United States, Argentina and the strain found in Colombia, also defining a clade of CanineCV sequences identified in foxes [Figure 3; (22)].

Phylogenetic analysis of CanineCV sequences identified in arctic and red foxes in Norway (23) showed a distinguishable clustering in five groups in the tree constructed with sequences of complete genomes and in the tree of concatenated amino acids of the Rep and Cap proteins. Group 1 included sequences identified in dogs, wolves and a badger found in Europe, Asia and the United States. Groups 2, 3 and 4 included sequences identified in dogs in Asia, and the sequence 09-10F from 2011 of a red fox in Italy, Group 5, was made up of sequences identified in foxes. The UCD3-478 strain did not fit into any of the previous groups (23). The presence of the sequence 09-10F/2011 in Group 4 indicates that the segregation of the groups does not occur based on the host where they were identified. The authors also define that the UCD3-478 strain may represent an intermediary between the groups 4 and 5 or the only sequence of a possible sixth group (23).

Recent analysis from Middle east including Turkish and Iranian samples belonging from diarrheic dogs through analysis of the partial Rep and Cap nt sequences, had found that the Iranian CanineCV strains were more closely related to strains detected in Turkey, allowing to hypothesize a possible introduction of the virus from this neighbor country (24–26). However, the most recent complete genome nucleotide sequences analysis of CanineCV strains obtained from Iranian dogs belongs to a separate independent clade (24).

Additionally, in 2020, the first systematic report on the use of codons and adaptation to the hosts of CanineCV was carried out in which all available sequences were used. The results suggest that CanineCV descends from the bat and that the sequences have been dominated mainly by natural selection, making it possible to propose a transmission hypothesis between species in which CanineCV may have evolved from bats (analysis of origin) and, subsequently, have adapted to domestic and wild canids (27).

## 4. Disease in dogs

In 2013, Li et al. characterized the complete genome of CanineCV from the liver of a dog with severe hemorrhagic gastroenteritis, granulomatous lymphadenitis, and vasculitis. Since then, CanineCV infection has been associated with clinical profiles of acute gastroenteritis, hemorrhagic diarrhea, signs of vasculitis, lymphadenitis, thrombocytopenia, neutropenia, and lymphopenia, which is related to immunosuppressive activity due to lymphoid tissue damage; the latter has also been described in infection by PCVs in pigs and in birds suffering Beak and Feather Disease Virus (BFDV) (3, 6, 28). Other authors have also associated CanineCV with respiratory signs such as dyspnea, nasal discharge, cough, or rales in the lungs (5). However, the virus has also been detected in samples of asymptomatic canines, so its pathogenesis and epidemiology cannot yet be clearly defined.

Different forms of infection could be suggested, such as subclinical, clinical or coinfection with other infectious agents, and the fecal route as the main route for transmission (3, 6, 28). With the objective of understanding and establishing the behavior of CanineCV infection, in addition to its prevalence and genetic diversity, multiple investigations have been carried out worldwide in dogs with symptoms compatible with those described and in apparently healthy dogs.

The prevalence of CanineCV in different countries, both in samples of patients with compatible signs and asymptomatic animals, is variable. Li et al. in 2013 in the United States (3) established a prevalence in stool samples of 11.3% in dogs with diarrhea, 6.9% in healthy dogs, and 3.3% in serum samples in dogs as signs of thrombocytopenia, neutropenia or history of ticks. The prevalence between positive samples from animals with diarrhea and samples from healthy dogs was not significant (3). In Asia, several studies have been conducted in different countries. In Taiwan, Hsu et al. (4) found a highly significant correlation between the presence of diarrhea and CanineCV, with a prevalence of 28.02% in dogs with diarrhea and 11.9% in healthy dogs; the authors suggested that the difference in the results with respect to the USA may be related to geographic distribution, molecular technique or primer design (4). In Northeast China, a prevalence of CanineCV of 15.6% in dogs with diarrhea and 6.7% in healthy dogs was found; also, authors found a significant difference between both prevalences (20).

Sun W et al. in 2019, in the southern region of Guangxi established a molecular prevalence of CanineCV in serum samples of dogs with diarrhea of 8.75% and in healthy dogs of 9.25% (10). In Germany, it was established a prevalence of CanineCV of 20.1% in dogs with diarrhea and 7.3% in healthy dogs, indicating that the virus can be found significantly more frequently in dogs with diarrhea (29). Nevertheless, no significant difference between the detection of CanineCV in healthy dogs and dogs with hemorrhagic diarrhea, with prevalence rates of 4.5 and 3.6%, respectively has been found also in Germany (28). In Vietnam, the evaluation for CanineCV was performed on 81 CPV-2c positive fecal samples, establishing that 19.8% of the samples were positive for CanineCV in diarrheic puppies less than 7 months of age (30).

In middle east region, the presence of CanineCv has been studied in Turkey and Iran. In Turkey, from a total of 150 rectal swab samples collected from animals with manifestations of gastrointestinal disease, nine positive samples were obtained (6%) with coinfection with the other two viruses between 2 to 5% (26). To investigate the prevalence and genomic characteristics of CanineCV in Iranian dogs, a total of 203 fecal samples from dogs were collected between February and November 2018 from five different geographical regions and examined by real-time PCR (qPCR). Thirteen dogs (6.4%) tested positive for CanineCV DNA, and all were detected in coinfection with CPV-2. Later in 2022 a second study was carried out in the same way using real-time PCR in 156 rectal swabs from clinically healthy canines. 14 positive samples for CanineCV were obtained (8.9% prevalence). The CanineCV circulation in non-diarrheal dogs in Iran highlighting the need for further epidemiological investigations (24, 25).

The only study reported to date on seroprevalence of CanineCV was conducted with samples taken between 2016 and 2017 in northeast China (31). Authors found a seroprevalence of 39.82%, with differences between the different cities ranging from 23 to 43%. Similarly, the study showed a significant difference in prevalence between dogs with diarrhea and healthy dogs (31). A compilation of the studies carried out to date in different countries and obtained from databases such as PubMed and Science Direct, with the purpose for the detection of CanineCV in domestic dogs is shown in Table 1.

**Table 1:** Studies conducted for the detection of CanineCV in domestic canines.

Country	Year	Type of sample	Clinical signs	Sampled animals	Reference
United States	2013	Stool, blood	Diarrhea, thrombocytopenia, neutropenia	35 sick 14 healthy	(3)
Italy	2014	Tissues	Hemorrhagic diarrhea	1 sick	(7)
United States	2016	Feces, tissues	Hemorrhagic gastroenteritis	3 sick	(6)
China	2016	Rectal swabs, feces	Diarrhea	58 sick, 19 healthy	(4)
Italy	2017	Rectal swabs, feces	Acute gastroenteritis	71 sick, 19 healthy	(32)
Germany	2017	Feces	Diarrhea	37 sick 6 healthy	(29)
Germany	2017	Feces	Hemorrhagic diarrhea	55 sick, 66 healthy, 54 CPV-2 positives	(28)
Thailand	2018	Oral or nasal swab, tissues	No	9 sick	(5)
China	2019	Serum	Diarrhea	81 sick 79 healthy	(10)
China	2019	Feces	Diarrhea	15 sick 3 healthy	(20)
United States	2019	Tissues	Hemorrhagic gastroenteritis, multisystemic vasculitis	1 sick	(33)
Argentina	2019	Tissues	Hemorrhagic diarrhea	3 sick	(21)
Turkey	2019	Feces	Diarrhea	150 sick	(26)
Colombia	2020	Feces	Hemorrhagic diarrhea	5 sick	(22)
Brazil	2020	Feces	Hemorrhagic gastroenteritis	1 sick	(16)
China	2020	Serum	Diarrhea	417 total	(27)
Vietnam	2021	Feces	Diarrhea	81 total	(30)
Iran	2022	Rectal swabs	No	156 total	(24)

The prevalence of CanineCV varies over a wide range. The differences between the studies may be due to multiple factors as mentioned before. In addition, it is not yet possible to define whether the virus is found more frequently in dogs with diarrhea than in healthy animals, as shown by the variations in the results of the reports published to date. Therefore, it is important to conduct molecular and research that clearly defines the epidemiological characteristics of the virus and its role as an infectious agent in gastrointestinal clinical disease in domestic dogs.

## 5. Histopathological findings

Necropsy and immunohistochemistry of retrospective cases of dogs with signs compatible with CanineCV has shown critical lesions, such as necrotizing vasculitis in the intestine and spleen, granulomatous lymphadenitis and histiocytic drainage in Peyer’s patches and lymph nodes, and microscopic lesions in the kidney (3). Clinico-Histopathological findings in a case report in Connecticut, United States, showed macroscopically pale mucous membranes, blue–black small intestine, black stools in the colon, splenomegaly, and multiple enlarged and hemorrhagic lymph nodes. At the microscopic level, circumferential transmural vasculitis and necrosis of crypt cells and lymphocyte necrosis in the lymph nodes and spleen were observed in the intestine, and vasculitis was also found in the liver, kidneys, lung, meninges, and brain (33). Similarly, lesions such as friability of the small intestine, focal congestion of Peyer’s patches, aqueous and hemorrhagic content in the small intestine, large intestine with multifocal petechiae and melena inside has been reported (6).

Histologically, loss of microvilli, necrosis in crypt cells, lymphoid necrosis in Peyer’s patches in the ileum, mesenteric lymph nodes and spleen, granulomatous inflammation, and increased histiocytes in lymph nodes were found (6). Compiling the above, it is possible to establish that the histopathological findings associated with CanineCV have a close similarity, both in the affected organs and in the lesions found, the most common being blue–black coloration in the intestine, lymphocyte necrosis and granulomatous inflammation. A similar histopathological profile has also been described in PCV2 infection in pigs; however, causality studies that demonstrate by *in situ* hybridization or immunohistochemistry the presence of this viral agent in compatible lesions are still lacking (33).

## 6. Infection in wild carnivores

Different studies have confirmed the presence of CanineCV in wild carnivores. A retrospective analysis from samples taken between 2013 and 2014, detected and molecularly characterized CanineCV in samples of organs from domestic dogs, wolves, foxes and badgers collected in regions of central and southern Italy; CanineCV was found in nine out of 34 wolves, eight out of 209 dogs, and one out of 10 badgers, confirming that the circulation of the virus is not restricted to domestic dogs (8).

CanineCV has been also confirmed in foxes in the United Kingdom, from samples taken from foxes suffering meningoencephalitis between 2009 and 2013. High frequencies of infection in histological samples and in serum of foxes measured bye by real-time PCR. Also, phylogenetic analysis revealed a close relationship with CanineCV identified in 2011 in the USA with an identity of 92 and 89% in the amino acid sequence in the Rep protein and in the nt sequence, respectively; however, the sequences of CanineCV from dogs and CanineCV from foxes grouped in separated clades (34). Notably, circovirus infection in wild foxes is associated with neurological signs, in contrast to domestic dog disease which was associated with gastrointestinal, respiratory and vasculitis. The fact that circoviruses that infect foxes and those that infect domestic dogs are very similar viruses but with different symptoms raises several questions for future research regarding the transmission and pathogenesis of both viruses between species of the same family (34). Also, seeking to evaluate the presence of CanineCV in red foxes (*Vulpes vulpes*) from Italy by using molecular and phylogenetic approaches, CanineCV DNA was detected in one of 32 processed stool samples; confirming that dogs and foxes could share phylogenetic related circoviruses (35).

In another retrospective analysis on samples of 51 arctic foxes (*Vulpes lagopus*) from the Svalbard archipelago, Norway and 59 red foxes (*Vulpes vulpes*) from Northern Norway (mainland) sampled from 1996 to 2018, CanineCV DNA was detected in 10 red foxes and 11 arctic foxes. Positive DNA were found even in samples from Artic foxes sampled in 1996, fifteen years before the first report in domestic dogs in USA, suggesting that wild carnivores could have harbored an ancestor of CanineCV that could later be transmitted to domestic canids, thus highlighting the role of wild carnivores in the transmission of pathogens to the domestic dogs populations (23).

## 7. Coinfection and comparative analysis to PCV

In the studies on the detection of CanineCV carried out to date, coinfection with other infectious agents has also been analyzed. Among these, the presence of bacteria such as *Salmonella* sp., *Campylobacter* sp. and enterotoxins of *Clostridium* sp.; parasites such as *Giardia* sp. and *Cryptosporidium* sp.; and viruses such as canine parvovirus type 2 (CPV-2), canine coronavirus and canine distemper has been found (3, 6, 7). In similar way, coinfection between several pathogens and circovirus has already been described in pigs, where it is relatively common (36). PCVs have been widely studied for several decades, and four different subtypes have been identified: PCV1, PCV2, PCV3 and PCV4 (36–38). PCV1 corresponds to a nonpathogenic type, described in 1974 as a contaminant in PK-15 pig kidney cell lines (39, 40). PCV2 is highly pathogenic, and it has been associated with a series of syndromes and pathologies classified into four forms of presentation that affect pig production worldwide, generating countless economic losses in the industry (41). PCV3 is associated with two clinical presentations called the systemic form and the reproductive form (42). Finally, PCV4 is the most recent discovery and has been reported only in China (43).

PCV2 was initially associated with multisystem wasting syndrome (PMWS); however, after multiple investigations, the association of PCV2 with other clinical conditions, such as hepatitis, reproductive failure, respiratory disease and nephropathy (PDNS), was established. It has also been associated with porcine respiratory complex disease (PRDC) (36, 38). Currently, it is recognized as the causative agent of some conditions known as porcine circovirus associated diseases (PCVAD), which mainly affect fattening pigs from 7 to 16 weeks of age, where there are clinical manifestations such as weight loss, hypertrophy of lymph nodes, jaundice, growth retardation, dyspnea, and abortions. Histopathological findings include lymphoid depletion, replacement by histiocytes in lymphoid tissue, bronchiolitis and interstitial pneumonia with mononuclear infiltrate in the lungs (41). There are several reports and studies on the role of coinfection with other infectious agents since it is unusual for PCV2 to generate clinical pictures as a single agent (36). It is generally found together with other microorganisms, such as *Salmonella* sp., *Mycoplasma hyopneumoniae*, swine influenza virus (SIV), porcine reproductive and respiratory syndrome virus (PRRSV) and porcine parvovirus (PPV). Coinfection has even been described between different PCV2 genotypes. Also, it has been suggested that coinfections are associated with a greater severity of infection (41, 44).

The adverse effect of PCV2 coinfections with the aforementioned agents could be explained by the repercussions on the immune system of pigs since the virus targets lymphoid tissues. The replication of PCV2 destroys the normal architecture of the lymphoid follicles, causing lymphoid depletion that is then replaced by histiocytes. Replication of the virus has been evidenced in organs such as the spleen, tonsils, lungs, kidney, liver and thymus; in the latter, PCV2 alters the selection of T lymphocytes, particularly in younger animals. It also infects dendritic cells, limiting their function; such effects generate severe lymphoid depletion and severe immunosuppression in pigs, increasing the probability of contracting other infections (41, 44).

There are also reports of the detection of PCV2 and PCV3 in dogs. To date, PCV2 has been discovered in other reservoirs, such as calves, goats, mice, and in carnivores, such as mink and foxes, indicating that transmission to nonporcine hosts is possible. Close contact between animals is probably the main route of transmission, particularly in multispecies farms and sites accessible to wildlife (45, 46). The reported studies of PCV2 in nonporcine hosts have associated the presence of the virus with gastrointestinal signs in dogs and with sterility in raccoons. However, the authors conclude on the need to continue with studies that allow defining the direct association between PCV2 and the clinical profile described. Similarly, the PCV3 genome has been detected in populations of domestic dogs, indicating its transmission to non-porcine hosts, and CPV/PCV3 coinfection has even been found in dogs (47, 48).

Similar to PCV2 infection, the pathogenesis of CanineCV and its participation as a coinfecting agent is not yet clearly defined. Current research suggests is that its role as a pathogen may be to exacerbate clinical conditions, like PCV2 infection, weakening the immune system and favoring the entry and dissemination of other viruses and bacteria. This is because, in the majority of the CanineCV detections performed, the virus has been found to be accompanied by other infectious agents, such as CPV with coinfection rates of up to 100% (22). To date, few reports confirmed CanineCV as a single agent in dog suffering acute gastroenteritis or hemorrhagic diarrhea (3, 21).

Based on the findings of the genetic material of CanineCV in lymphoid tissues, it has been proposed different hypotheses regarding the clinical manifestation of the CanineCV infection (6, 28). First, the replication of CanineCV in lymphoid tissues could be favored by previous infection with CPV, without apparent clinical manifestation or generation of disease. Second, the presence of a synergistic effect where CanineCV infection causes immunosuppression and allows the development of the clinical disease by CPV, even in vaccinated individuals, a situation that has been described in pigs as mentioned above.

It has been also evaluated the possible role of CanineCV in acute hemorrhagic diarrhea syndrome. According to the results, it has been reported a low probability that CanineCV acts as a putative agent of the syndrome, but CanineCV may be a predisposing element for bacterial or viral infections. The prevalence of CanineCV in the study was 4.6%, indicating that it could be only an incidental finding where the virus would be part of the normal intestinal microbiota of canines (28); this has also been described in pigs where PCV1 is present in asymptomatic animals as a nonpathogenic agent (49). The most significant finding of the study was the finding of a higher mortality in dogs that presented CPV2/CanineCV coinfection. There are previous reports where a greater susceptibility or increased severity of infection was found when CPV2 manifests in coinfection with other pathogens, such as enteric coronavirus. The authors suggest that the same can happen with CanineCV, as has been observed and reported in pigs in PCV2 infection.

## 8. Final remarks

The discovery of emerging viruses such as CanineCV raises an important research topic in domestic and wild animals. The evidence of multiple gaps in the knowledge of this virus in aspects such as pathogenic and epidemiological characteristics raises the need to create and continue with studies that allow expanding and defining the knowledge about CanineCV infection. Scientific evidence supports that this virus poses a risk to the health and well-being of the canine population, so work must be done to define many concepts and gaps in the understanding of this emerging pathogen. Therefore, its necessary to strength the knowledge of veterinarians and clinicians about the possible manifestations as a disease not only as a single agent but also in coinfection with other pathogens. Additionally, the role of wild canids should be highlighted, since, according to recent research, it is evident that in this population, CanineCV seems to be more pathogenic, and its participation in the transmission of the virus to domestic canines seems to be relevant, especially because human invasion of wild habitats favors contact between both populations, which contributes to the transmission of interspecies pathogens.

**Figure 4:**
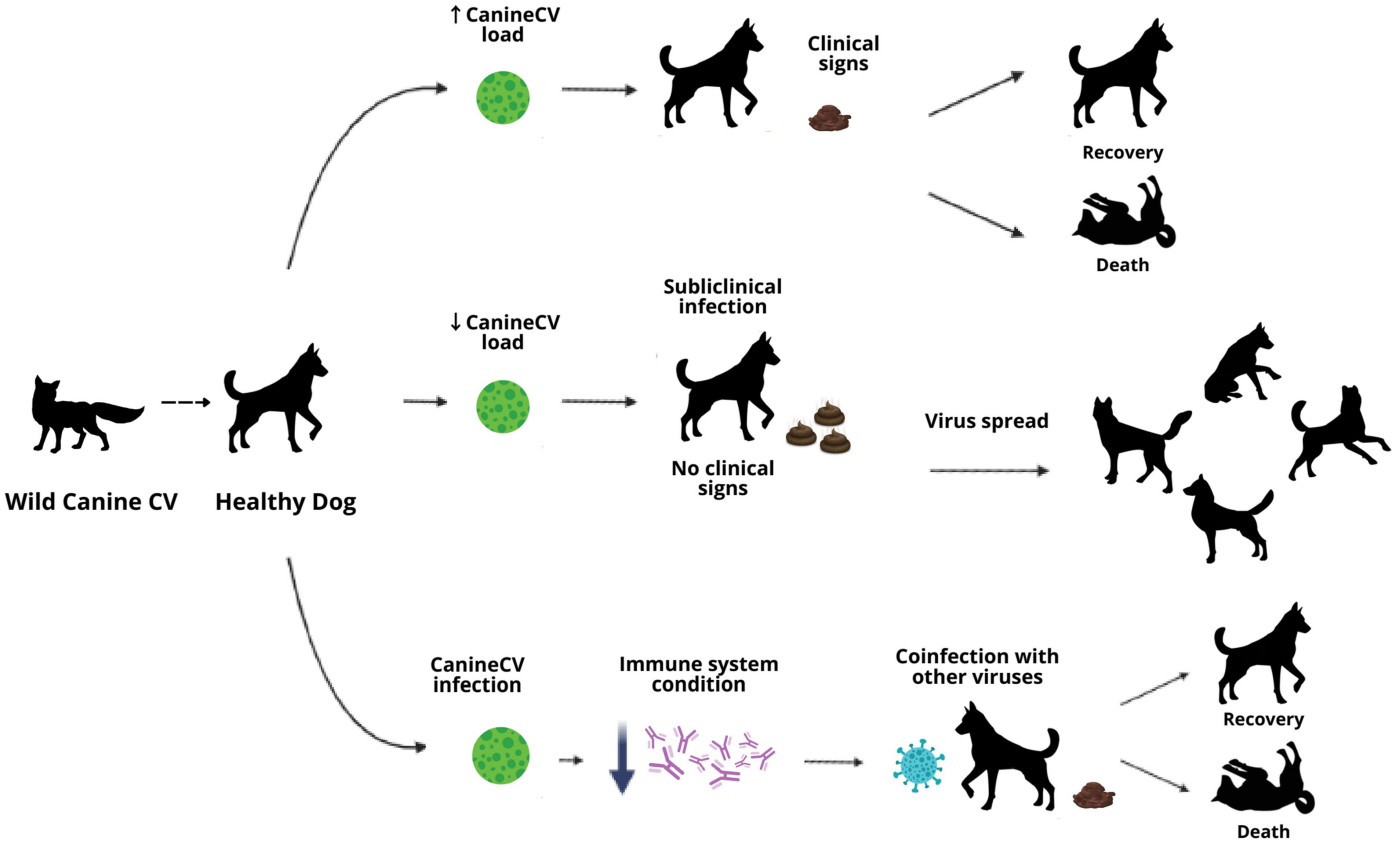
Diagram of possible disease routes in CanineCV infection in domestic dogs.

The role of CanineCV as a primary agent or coinfecting in enteric diseases in canines remains uncertain; existing research suggests the importance of continuing with other study models that provide new information to define the concepts that remain doubtful until now. As can be observed in the development of this article, CanineCV could have different pathways of disease development in canines, either as a primary agent that leads to generating the clinical signs described or to cause subclinical infection where there are no signs but where the animal could spread the virus or contribute to the severity of the disease in coinfection with other agents. In addition, CanineCV, like the other circoviruses, could play an important role in the immune response of the animal, favoring the entry of other infectious agents or preventing its recovery (Figure 4).

## 9. Conclusion

The pathogenic and epidemiological characteristics of CanineCV are currently poorly understood, so CanineCV represents a multidisciplinary exploration challenge both in the field of research and in the clinic. Understanding the particularities of this new virus either in healthy animals or coinfected with other agents could provide valuable information regarding prophylactic or therapeutic practices in enteric diseases in canines, contributing to the health and well-being of canine populations. Thus, it is necessary to continue carrying out research in different canine populations that allow the evaluation of the epidemiology of CanineCV in geographical sites not yet explored and its behavior in healthy or coinfected animals, in addition, to monitoring the clinical evolution of the infected animals to help to understand the pathogenesis of the CanineCV.

## Author contributions

JR-S and JJ: conceptualization, validation, resources, and data curation. DG-B and DV-B: methodology, formal analysis, and investigation. DG-B: writing—original draft preparation. DG-B, DV-B, SG-R, JJ, and JR-S: writing—review and editing. SG-R: software and visualization. JR-S: supervision, project administration, and funding acquisition. All authors contributed to the article and approved the submitted version.

## Funding

This research was funded by a CONADI-UCC grant to JR-S and the APC was funded by CONADI-UCC.

## Conflict of interest

The authors declare that the research was conducted in the absence of any commercial or financial relationships that could be construed as a potential conflict of interest.

## Publisher’s note

All claims expressed in this article are solely those of the authors and do not necessarily represent those of their affiliated organizations, or those of the publisher, the editors and the reviewers. Any product that may be evaluated in this article, or claim that may be made by its manufacturer, is not guaranteed or endorsed by the publisher.

## Footnotes

^1^ http://www.ictvonline.org/virustaxonomy.asp
